# Feasibility of testing the effectiveness of a theory-informed intervention to reduce imaging for low back pain: a pilot cluster randomised controlled trial

**DOI:** 10.1186/s40814-022-01216-8

**Published:** 2022-12-09

**Authors:** Hazel J. Jenkins, Simon D. French, Anika Young, Niamh A. Moloney, Chris G. Maher, John S. Magnussen, Blake F. Dear, Mark J. Hancock

**Affiliations:** 1 Faculty of Medicine, Health and Human Sciences, Macquarie University, Level 2, 75 Talavera Rd, Sydney, 2109 Australia; 2 The University of Sydney, Sydney Musculoskeletal Health, Sydney, Australia

**Keywords:** Low back pain, Diagnostic imaging, Medical imaging, General medical practice, Patient education, Pilot study, Decreasing imaging overuse

## Abstract

**Background:**

General medical practitioner (GP) recruitment and subsequent data collection in clinical practice are challenging and may limit successful completion of a large-scale trial. The aim of this study was to assess the feasibility of undertaking a cluster randomised controlled trial to test an intervention to reduce non-indicated imaging for low back pain in general medical practice.

**Methods:**

A pilot cluster randomised controlled trial was performed, with recruitment of GPs and randomisation of GP clinics. All GPs attended a training session and were asked to record low back pain codes in electronic medical records for any low back pain presentations. Intervention group GPs were trained in the use of a patient education booklet to be used during low back pain patient visits. Control group GPs provided usual care. Outcomes for the proposed trial were collected to determine feasibility. GP recruitment was assessed as the proportion of GPs approached who consented to participate. Low back pain imaging outcomes were collected from electronic medical records (counts of patients presenting with low back pain) and from Australian healthcare administrative (Medicare) data (counts of imaging use). GP compliance with study procedures was assessed and qualitative data reported.

**Results:**

Thirty-four GP clinics were approached, with four participating (12%). At these clinics, 13/19 (68%) GPs consented to participate, and 10/19 (53%) started the study. Outcome data were collected from medical records for all GPs. Three GPs (30%) withdrew consent to access Medicare data, limiting reporting of imaging outcome measures. Three GPs (30%) self-reported low compliance entering low back pain codes.

**Conclusions:**

This pilot cluster randomised controlled trial demonstrated the feasibility of many aspects of a full-scale effectiveness study, while also identifying a number of challenges that need to be resolved. Recommendations related to GP recruitment, study compliance, data collection, and outcome measures were made to increase the success of a future trial.

**Trial registration:**

Australia and New Zealand Clinical Trials Register (ANZCTR), Trial ID: ACTRN12619000991112; Registered 11 July 2019, https://www.anzctr.org.au/Trial/Registration/TrialReview.aspx?id=376973

**Supplementary Information:**

The online version contains supplementary material available at 10.1186/s40814-022-01216-8.

## Key messages regarding feasibility

- The feasibility of recruiting general medical practitioners (GPs) and collecting low back pain imaging data in primary care is currently uncertain.
- The percentage of GP clinic recruitment was low (12%); however, within the recruited clinics practitioner recruitment was high (68%). Outcome data was successfully extracted, but relies on practitioner use of codes.
- Strategies to reach a large number of clinics or facilitate in-person contact with practitioners need to be considered for successful recruitment. Audits of practitioner use of codes are needed to assess accuracy of data collection.

## Background

It is widely accepted that imaging is overused in the management of low back pain (LBP) [1, 2], and has not decreased over time despite this knowledge [3]. Overuse of imaging for LBP has been associated with increased downstream healthcare use, e.g. further imaging and other investigations, increased surgery and other treatment, and potentially poorer outcomes for patients [1, 4, 5]. Previous strategies to reduce non-indicated imaging use do not show strong evidence of effect [6]. Many barriers to decreasing the use of imaging for LBP have been identified [7], and addressing these barriers remains a challenge.

A theory-informed intervention to reduce non-indicated imaging for LBP has been developed to address two behaviours contributing to the overuse of imaging in general medical practice: (1) pressure from patients to refer for imaging; and (2) general practitioners (GPs) using imaging referrals to reassure patients with LBP and to quickly acknowledge their concerns. The development of this intervention has been described previously [8]. The behaviours that were addressed by the intervention were selected as they are commonly reported barriers to reducing imaging referral for LBP [8] which have not been adequately addressed with previous interventions [6]. This intervention comprises: (1) a LBP management booklet for GPs to use during a patient consult to reinforce GP decision-making and facilitate GP-patient communication; and (2) GP education and training in using the booklet. Preliminary testing of the booklet in Australia and Finland showed that the intervention was acceptable to both GPs and patients and thought likely to be helpful in the management of LBP [8, 9]. The effectiveness of the booklet to decrease LBP imaging has not been tested.

To test the effectiveness of the intervention, an accurate and feasible measure of GP imaging referral behaviour is required. Previous studies have reported lumbar imaging use as counts of imaging use (with no denominator) [10], proportions of imaging use with total number of patient presentations (with any complaint) as the denominator [11], or proportions of imaging use with the number of LBP presentations as the denominator [12]. It is currently unclear whether there are meaningful differences in results when different outcome measures of imaging use are reported.

Collecting data on the number of LBP presentations in primary care can be challenging. Recruiting LBP patients has met with substantial challenges in general practice, particularly when GPs were not involved in the recruitment process to avoid selection bias [13, 14]. Previous studies [15, 16] have avoided issues related to patient recruitment by collecting routinely recorded electronic medical data on patient diagnosis (e.g. ICD-10 codes [17]). However, this is challenging in healthcare systems, such as in Australia, where electronic medical data collection, while possible, is not regulated, so diagnostic codes are not consistently recorded for patients presenting with LBP in primary care. It is not known whether GPs will consistently use LBP codes in medical record software to enable extraction of data on the number of patients with LBP presenting for care. In Australia, imaging referral information is routinely collected by public healthcare (Medicare) systems; however, GPs need to provide consent to access these data. GP recruitment for clinical practice research is often challenging [18], and it is possible that requesting GP consent to access data from medical records may be a further challenge to recruitment.

The overarching aim of this study was to test the feasibility of key study design and processes planned for a future large-scale effectiveness trial. Four specific aims were identified to assess the specific processes to be tested:

- Aim 1: To assess the feasibility of recruiting eligible GP practices and practitioners with a spread of demographic characteristics
- Aim 2: To assess GP compliance with study processes, including attendance at the training session and use of electronic medical record codes
- Aim 3: To assess the feasibility of collecting counts of (i) lumbar imaging; (ii) LBP patient presentations; and (iii) total patient presentations to allow calculation of measures of lumbar imaging use
- Aim 4: To explore if different measures of quantifying imaging use (i.e., counts, proportion of LBP presentations, proportion of total patient presentations) produce differences in within-group results.

## Methods

A pilot cluster randomised controlled trial was conducted in general medical practices in Sydney, Australia. A cluster design is important for future large-scale effectiveness testing; randomising practitioners at the level of the practice limits contamination between intervention and control groups. This paper is reported in accordance with the CONSORT 2010 extension to randomised pilot and feasibility trials (checklist available in Additional file 1) [19]. Ethics approval was granted by Macquarie University Human Research Ethics Committee, reference number: 5201949847695. The study was prospectively registered through the Australia and New Zealand Clinical Trials Register (ANZCTR), Trial ID: ACTRN12619000991112.

### General practitioner recruitment

GPs were recruited by approaching local general medical practices (clinic contact) and GPs known to the research team (in-person contact). To be eligible to participate, the GP needed to use electronic medical record software with the ability to search for treatment codes (e.g. Medical Director or Best Practice software), see patients with LBP, work for a minimum of 10 h per week, work in a practice with at least one other GP, and consent to collection of data describing imaging referrals (using Medicare Benefits Schedule (MBS) data) and LBP patient presentations (using electronic medical record data).

A member of the research team met with interested GPs to explain the study and obtain consent. If one GP consented to participate, all other GPs in their practice were also invited to participate. At least two GPs from each practice were required to participate.

### Randomisation

Randomisation was performed at the level of the medical practice using a computer-generated list of random numbers with equal allocation. Randomisation was performed centrally by a researcher not involved in practitioner recruitment or assignment, and allocation for the medical practice was concealed until all practitioners willing to undertake the study had consented. After practitioners were informed of randomisation, no further practitioners from that clinic were recruited to the study. Blinding of participants or the research team could not be performed.

### Intervention

The intervention comprised a patient education booklet (available in both hardcopy and digital formats) that is designed to be used by the GP during a patient consult, plus a 20-min GP training session about the use of the booklet. The development and preliminary testing of the booklet has been previously described [8] and a copy of the booklet and the training resources are available online [20]. Training was conducted by a member of the research team (HJ). The training session included: discussion of the appropriate use of imaging for LBP; introduction to the development of the booklet and its purpose; explanation of the purpose and how to use the different elements in the booklet; suggestions for integrating use of the booklet within a consult; and a demonstration of use by the training facilitator (Additional file 2). Participants were also provided with a pre-recorded video of the training session [20], an information sheet on the booklet [20], and publications regarding the appropriate use of imaging for LBP [21–23]. GPs were asked to use the patient education booklet with any patients presenting with LBP, as they thought appropriate, for the 4-month study period.

### Control

GPs in the control group were asked to provide usual care over the 4-month study period. Access to the intervention was provided to the control group at the completion of the study period.

### Study processes

GPs in both intervention and control groups completed an initial written questionnaire and received a 10-min face-to-face training session outlining the study processes. During this training, all GPs were instructed to record all LBP presentations over the 4-month study period using one of eight pre-determined LBP codes within the medical record software. For GPs in the intervention group, the study processes training session was held at the same time as the intervention training (Additional file 2).

At the end of the 4-month study period, all GPs were asked to complete a face-to-face, Zoom, or telephone semi-structured interview with one of the research team regarding their experience during the study.

At the conclusion of the study, GPs in the intervention group were provided with an AU$100 gift voucher for their time in attending the 30-min training session and completing the post-study interview. GPs in the control group were provided with an AU$50 gift voucher for their time in attending the 10-min training session and completing the post-study interview. At the conclusion of the study, participating GPs were provided with a personalised summary of their lumbar imaging use during an 8-month period (4 months before and 4 months after training) and a certificate of completion which could be used as evidence for continuing professional development.

### Data collection

#### GP recruitment data (aim 1)

We recorded the number of GP clinics and GPs who (i) were initially approached (including method of approach); (ii) requested additional information; (iii) met with the research team; (iv) consented to participate; (v) met eligibility criteria; and (vi) entered the study. The initial questionnaire (Additional file 3) included demographic data (e.g. years in clinical practice, sex, special interest in LBP) and questions about the GPs’ beliefs of the usefulness of imaging for LBP (Likert scale, strongly agree to strongly disagree).

#### GP compliance with study processes data (aim 2)

We recorded the number of GPs who (i) attended the allocated training session; and (ii) provided access to Medicare and electronic medical record data. The post-study interview (Additional file 4) included Likert scale and open-ended questions on compliance with the study processes, including (i) their ability to use the necessary LBP codes in the electronic medical record (all GPs); and (ii) acceptability of the intervention training session (intervention group GPs). Interviews were recorded and transcribed by one of the researchers (AY).

#### Imaging and patient presentation outcome measure data (aims 3 and 4)

Data on patient presentations and imaging referrals were collected for an 8-month period: 4-months before and after the training session.

Counts of lumbar imaging (X-ray, CT, or MRI) were collected from Australian Medicare data for each participating GP. No patient identifying information was provided.

The number of patient presentations were collected using two methods. Counts of patient consultations (for any complaint) for each GP was collected from Australian Medicare data, as outlined above. Counts of LBP patient presentations for each GP were collected from clinic electronic medical records. No further patient details were extracted. Data extraction was performed by one of the researchers (AY) or an administrative staff member from the clinic, trained in the data collection protocol.

### Data analysis

#### GP recruitment (aim 1)

The proportion of GP clinics and GPs recruited from the number initially approached was reported with 95% confidence intervals at each stage of the recruitment process. Results were stratified for the method of initial approach: through the clinic practice manager (clinic) or through GPs known to the research team (in-person). Demographic characteristics, imaging beliefs, baseline number of LBP patients seen per month, and number of GPs with low (< 10%), moderate (10–30%), and high (> 30%) use of imaging (calculated from the data obtained for the 4 months prior to training delivery) were described to assess the characteristics of recruited GPs.

#### GP compliance with study processes (aim 2)

Qualitative interview data were coded in NVivo 12 plus by two of the researchers (HJ and AY). Data were analysed using framework thematic analysis [24] to describe: (i) self-reported GP compliance with entering LBP codes in the electronic medical records; (ii) associated barriers or facilitators to entering LBP codes; and (iii) acceptability of the intervention group training session. The initial thematic framework was determined by consensus between two of the researchers (HJ and AY), with final analysis discussed and agreed upon by the research team. The proportion of GPs attending the initial training session was reported quantitatively.

#### Collection of imaging use outcome data (aim 3)

To assess the feasibility of data collection for the main trial, the sample size for this pilot study was set at a minimum of four GP clinics with at least two practitioners in each clinic, based on an estimated 10 new LBP presentations per GP during each 4-month study period [25, 26].

Counts of imaging use, LBP presentations, and total presentations were reported per GP per month of the study period. Monthly proportions of imaging use per LBP presentations and per total patient presentations were calculated.

#### Exploring trends in results using different outcome measures (aim 4)

Three different summary measures of imaging use were calculated for each month of the 8-month study period: (1) the proportion of lumbar imaging divided by the number of patients presenting with LBP; (2) the proportion of lumbar imaging divided by the number of patients presenting with any condition (reported per 1000 patients); and (3) counts of lumbar imaging (no denominator). For each outcome measure, the proportion of imaging use per month compared to imaging use in month 1 was calculated to standardise the results and enable comparison between the three different outcome measures.

## Results

### GP recruitment (aim 1)

**Fig. 1:**
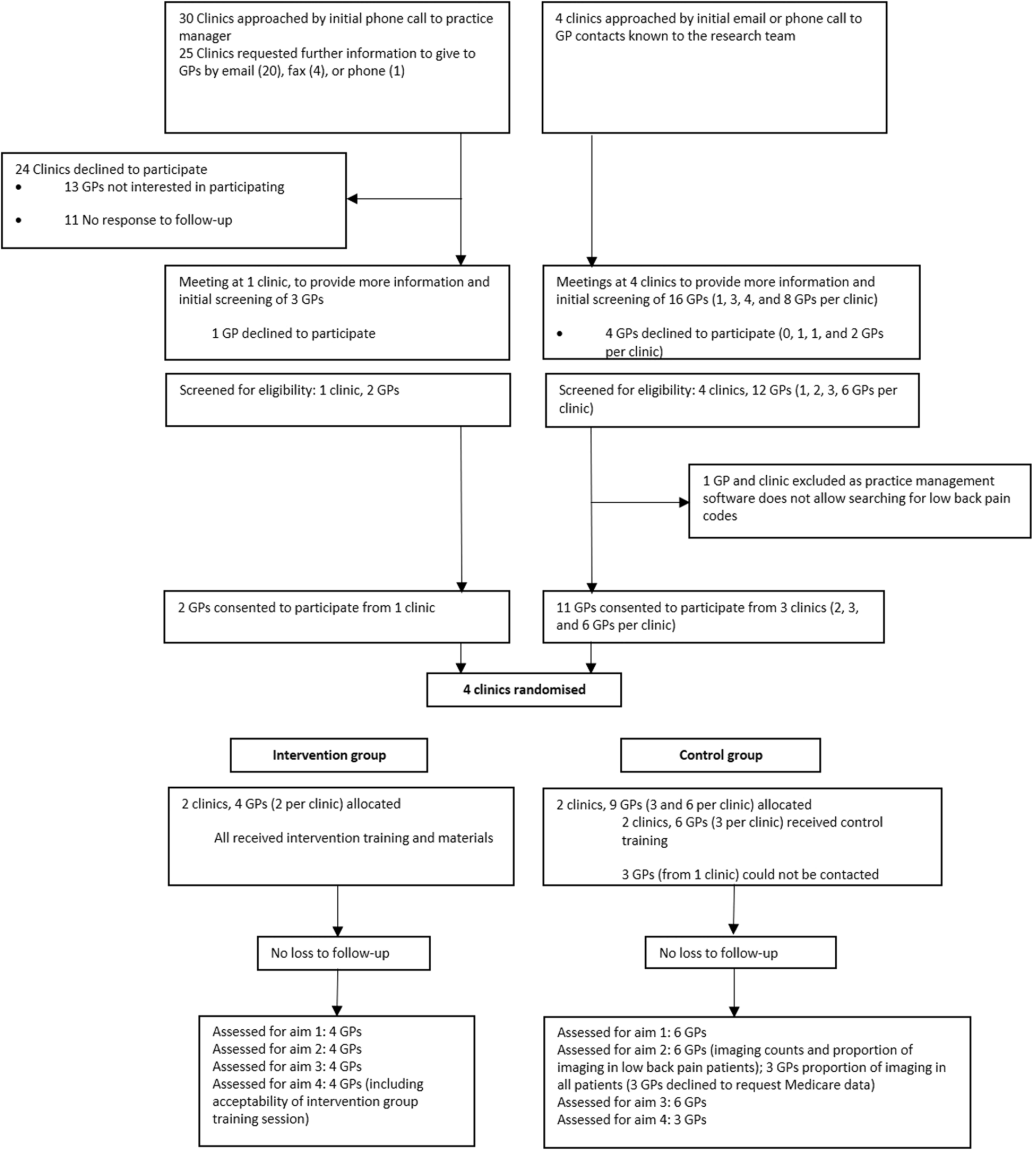
Study flow diagram

GP recruitment was performed between July 2019 and January 2020 as shown in the study flow diagram in Fig. 1. The proportion of GPs recruited at the different stages and different modes of contact are presented in Table 1. In total, 34 medical practices were approached for inclusion in the study, with four practices meeting inclusion criteria and randomised (12%). Nineteen GPs from five medical practices met with the research team for further information, with 13 GPs consenting to participate (68%). Direct initial contact with GPs resulted in more successful recruitment than approaching through the practice manager, with 75% of practices and 85% of GPs recruited through this method. Three of the 13 GPs who initially consented to participate in the study did not respond to requests to organise a time for the training session, consequently 10 GPs from four practices started the study (53% of those who received information about the study). Although the clinic had been randomised, the three GPs who could not be contacted had not been informed of their group allocation.

**Table 1:** GP recruitment and training completion

	In-person contact (*N* = 4 clinics)	Clinic contact (*N* = 30 clinics)	Overall (*N* = 34 clinics)
Received written or verbal study information (advertisement)	4/4 (100%)	25/30 (83%)	29/34 (85%)
Clinics and GPs meeting to receive more study information	4/4 clinics (100%) 16 GPs	1/25 clinics (4%) 3 GPs	5/29 clinics (17%) 19 GPs
Number of clinics and GPs meeting inclusion criteria	3/4 clinics (75%) 15/16 GPs (94%)	1/1 clinics (100%) 3/3 GPs (100%)	4/5 clinics (80%) 18/19 GPs (95%)
Number of GPs meeting inclusion criteria consenting to participate	11/15 GPs (73%)	2/3 GPs (67%)	13/18 GPs 68%
Number of consenting GPs attending training session	8/11 GPs (73%)	2/2 GPs (100%)	10/13 GPs (77%)

Demographic characteristics of the ten included GPs are presented in Table 2, showing recruited GPs of different sex, years in clinical practice, special interest in LBP, and baseline lumbar imaging referral. Most GPs reported confidence in managing LBP and did not agree that imaging is generally useful in the management of LBP presentations. Baseline differences were evident between intervention and control group GPs, with GPs allocated to the control group having fewer LBP presentations and lower imaging use in the 4 months period prior to delivery of the training session.

**Table 2:** GP baseline characteristics

	Overall (*N* = 10)	Intervention (*N* = 4)	Control (*N* = 6)
Sex (female *N*, %)	4 (40%)	1 (25%)	3 (50%)
Years in practice			
< 10 years	4 (40%)	2 (50%)	2 (33%)
10–19 years	3 (30%)	0 (0%)	3 (50%)
> 20 years	3 (30%)	2 (50%)	1 (17%)
Special interest in back pain	5 (50%)	1 (25%)	4 (67%)
Continuing education in back pain	5 (50%)	1 (25%)	4 (67%)
Confident in ability to manage back pain			
Agree	8 (80%)	4 (100%)	4 (67%)
Neutral	2 (20%)	0 (0%)	2 (33%)
Disagree	0 (0%)	0 (0%)	0 (0%)
Patient pressure to refer for imaging			
Agree	4 (40%)	0 (0%)	4 (67%)
Neutral	4 (40%)	3 (75%)	1 (17%)
Disagree	2 (20%)	1 (25%)	1 (17%)
Imaging is useful in the management of low back pain			
Agree	0 (0%)	0 (0%)	0 (0%)
Neutral	3 (30%)	1 (25%)	2 (33%)
Disagree	7 (70%)	3 (75%)	4 (67%)
Likely to refer for imaging for low back pain			
Agree	1 (10%)	0 (0%)	1 (17%)
Neutral	0 (0%)	0 (0%)	0 (0%)
Disagree	9 (90%)	4 (100%)	5 (83%)
Baseline monthly low back pain patients (mean, SD)	49.5 (10.0)	41.3 (9.1)	8.3 (1.7)
Baseline lumbar imaging use			
Low imaging use (< 10%)	4/10 (40%)	0/4 (0%)	4/6 (67%)
Moderate imaging use (10–30%)	1/10 (10%)	0/4 (0%)	1/6 (17%)
High imaging use (> 30%)	5/10 (50%)	4/4 (100%)	1/6 (17%)

Special interest in back pain: yes/no response to ‘do you have a special interest in back pain as a general practitioner?’; continuing education in back pain: yes/no response to ‘Have you done any continuing education in back pain in the last 2 years?’; confident in ability to manage back pain: 5-point Likert scale response from strongly disagree to strongly agree to ‘I feel confident in my ability as a general practitioner to manage patients with low back pain’; Patient pressure to refer for imaging: 5-point Likert scale response from strongly disagree to strongly agree to ‘I feel pressure from patients to refer for imaging (X-rays, CT, MRI) for low back pain’; Imaging is useful in the management of low back pain: 5-point Likert scale response from strongly disagree to strongly agree to ‘Imaging (X-rays, CT, MRI) of the lumbar spine is useful in the workup of patients with acute low back pain’; likely to refer for imaging of low back pain: 5-point Likert scale response from strongly disagree to strongly agree to ‘I am likely to order imaging (X-rays, CT, MRI) for acute low back pain’; All Likert responses divided into agree (strongly agree or agree), neutral, or disagree (disagree or strongly disagree) for analysis; baseline monthly low back pain patients calculated from the average number of monthly low back pain presentations for the 4 months before training; baseline lumbar imaging use calculated as the average monthly proportion of lumbar imaging divided by the number of low back pain presentations for the 4 months before training

### GP compliance with study processes (aim 2)

#### GP compliance using LBP codes

GP compliance with using LBP codes and barriers and facilitators to use are presented in Additional file 5. Self-reported compliance entering LBP codes into the electronic medical records was generally high, with 70% (7/10) reporting using the codes all or most of the time and only 30% (3/10) reporting that they used the codes less consistently. GPs generally found the codes easy to use. Facilitators of use included already using medical record codes, having a leadership role in the medical practice, and being involved in the study. GPs reported forgetting to use the codes because of lack of time within the consult, infrequent LBP presentations, or LBP being a secondary patient complaint.

#### GP training completion and acceptability

GP training completion and qualitative responses on the acceptability of the training session are presented in Additional file 5. GP training was conducted between September 2019 and January 2020. All GPs who started the study attended an in-person training session. Three of the four GPs in the intervention group (75%) reported that the training session was useful to get initial instruction on how to use the intervention and receive reminders and reinforcement on managing LBP. An in-person session (as opposed to an online training module) was seen as important to give an opportunity to ask questions and clarify the use of the intervention. One GP (25%) did not find the training useful, reporting that they did not remember the session well and felt it was presented too quickly as it was scheduled during their working day between patient visits.

### Collecting imaging use outcome data (aim 3)

#### Counts of imaging

Additional file 6 shows the monthly counts of lumbar imaging per GP. Counts of imaging were successfully collected for seven of the included GPs using Medicare data. Three GPs from one clinic withdrew their consent to request Medicare data during the study due to their concerns that requesting data may lead to a higher risk of future Medicare audits; this was despite being provided with information from Medicare that audits are not triggered by requests for data. For this clinic, de-identified counts of lumbar imaging data were collected from the electronic medical record by clinic staff.

#### Counts of LBP presentations

Monthly counts of LBP presentations per GP were able to be successfully collected from electronic medical records as shown in Additional file 6. One GP, who also reported low use of LBP medical record codes, had no counts of LBP patients during either study period.

#### Counts of all patient presentations (for any complaint)

Additional file 6 shows the monthly counts of patient presentations (for any complaint) per GP for seven of the included GPs using Medicare data. Total patient presentations were not collected for the three GPs who withdrew their consent to request Medicare data.

### Exploring trends in results using different outcome measures (aim 4)

**Fig. 2:**
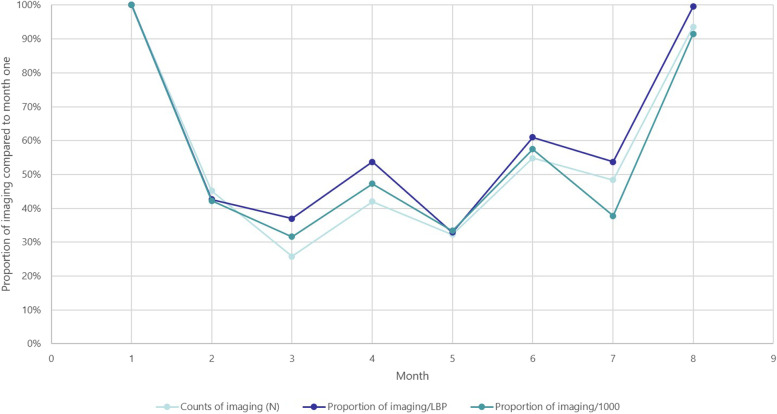
Monthly summary outcome measures of lumbar pain imaging

The three summary outcome measures of lumbar imaging are presented in Additional file 6 and the proportion of imaging use compared to month one is presented in Fig. 2. The magnitude of each outcome measure differs markedly (Additional file 5); however, monthly trends over time of increasing or decreasing imaging use are relatively consistent between outcome measures (Fig. 2).

## Discussion

This study piloted the study design and processes planned for a large-scale cluster randomised controlled trial to test the effectiveness of an intervention designed to reduce imaging for LBP. The study demonstrated feasibility of many aspects of a full-scale effectiveness study, while also identifying a number of challenges that need to be resolved.

### GP recruitment (aim 1)

The proportion of GPs recruited through initial contact with the clinic was low, with only one clinic from 30 contacted (3.3%) requesting a visit from the research team, and two GPs recruited. This is consistent with results from a large-scale cluster trial in a similar setting, where 5% of approached clinics were recruited [11]. In contrast, initial in-person contact with the GP showed a higher proportion of recruitment, with all GPs initially contacted meeting with the research team, and approximately two-thirds of GPs who met with the research team consenting to participate (13/19, 68.4%). Previous studies [18], finding similar challenges in GP recruitment have suggested different in-person methods of contacting GPs, such as at conferences, clinical practice meetings, and using connections such as primary healthcare networks, which should be considered moving forward.

The spread of demographic characteristics of the recruited GPs was reasonable, especially given the small sample size recruited. GP demographics were comparable to similar studies with Australian GPs [11, 25, 27], except that GPs in this study tended to have a greater interest in LBP [11, 27]. In the current study, most GPs reported confidence in their ability to manage LBP and disagreed that imaging is useful in most cases of LBP management. In contrast, a lack of confidence in managing LBP has been reported as a barrier to appropriate management of LBP and this was a target behaviour of the developed intervention [8]. Future recruitment strategies may need to target GPs with characteristics who are more likely to find the intervention useful. Differences between baseline characteristics in intervention and control groups were evident, but likely reflective of the cluster design and the small number of GPs randomised. Similar imbalances have also been noted in previous large-scale cluster trials [11].

### GP compliance with study processes (aim 2)

#### GP compliance using LBP codes

Using electronic medical record data to obtain outcome measures of imaging use has been successfully used in previous studies within healthcare systems with regulated use of patient diagnosis codes [15, 16]. In contrast, in this study, GPs were asked to record LBP codes that they may not normally use. Most GPs reported high compliance with using LBP codes; however, these were self-reported data and may be associated with bias. Some GPs reported forgetting to use codes, and low compliance with using the LBP codes would strongly impact the accuracy of counts of LBP patients presenting and the subsequent measure of imaging use calculated. Regular reminders to GPs to use codes in the practice management software may help to improve this behaviour for future studies [28]. An audit of a randomised selection of clinical records is recommended in future studies to assess GP compliance.

#### GP training completion and acceptability

The face-to-face intervention training session was completed by all GPs, but dedicated time and ongoing support are needed to improve acceptability, with one GP (25%) reporting that the training session was too rushed. Most of the intervention group GPs found the training session useful, particularly in the face-to-face format. GPs reported that they preferred an initial face-to-face training session over a pre-recorded or online version, but subsequent access to pre-recorded or online options would be useful as reminders. French et. al. [11] used longer and more structured training workshops to educate GPs; however, only 61% of GPs were able to attend the workshops, with the remainder having poor uptake of recordings of the information sessions. Therefore, a balance needs to be achieved between the time demands of the training session and GP attendance.

### Collecting imaging use outcome data (aim 3)

Data for each GP for the 8-month study period were successfully collected from clinic electronic medical records; however, originally piloted data collection strategies needed to be modified to ensure that all LBP presentations collected were within the designated study period. While the data collection method was still feasible, the modified method was more resource intensive, which needs to be considered in future trials.

We were able to successfully capture deidentified imaging count data from Medicare, a method that has also been used in other studies conducted in the Australian healthcare setting [11, 29]. Some limitations were identified that should be addressed in the future large trial. Imaging data are only collected by Medicare when the patient uses an imaging referral. Therefore, imaging referrals that are provided but not used cannot be captured and some imaging use may be recorded in a different month to the patient visit. These limitations will be less significant if data are collected over a longer time-frame, and this should be considered for future studies. One clinic (three GPs) declined to request Medicare data at the end of the study. Although imaging data were alternatively collected from the electronic medical record for this clinic, the process was resource intensive and may not be feasible for a large-scale trial. GPs declining to access Medicare data would need to be withdrawn from the study, and this should be considered in sample size calculations. Better training and explanation of the trial processes at the start of the study may also help to decrease this limitation. Medicare only collects data on imaging rebated within the Australian public healthcare system. This includes the majority of lumbar X-ray and CT referrals; however, GPs can only refer for Medicare-rebated MRIs for very limited clinical indications. Despite this limitation, it is likely that Medicare data will capture the majority of imaging use as GPs are also less likely to refer for MRI due to the lack of Medicare rebate, with MRI referred for in less than 0.05% of all imaging referrals [30]. Private referrals for lumbar imaging will not be captured using the processes in this study, and while these referrals are likely to be small, the generalisability of results to other regions needs to be considered.

### Exploring trends in results using different outcome measures (aim 4)

Although absolute values were significantly different, when the outcomes were standardised to the proportions of imaging use compared to month 1, similar trends in results were seen with all measures of lumbar imaging use. Logically, calculating the proportion of imaging among presenting LBP patients is more accurate, and more representative of the population of interest, than calculating the proportion among all presenting patients (for any complaint). However, extracting data on LBP presentations is more resource intensive, data accuracy may be limited by GP compliance with using LBP codes, and there is increased risk of selection bias [31]. Measuring the proportion of imaging among patients presenting for any complaint may be a useful proxy measure that is easily collected and has been used in previous studies [11]. Sample sizes may need to be substantially larger to detect statistically significant differences in imaging use. Where possible we suggest that the proportion of imaging use in LBP presentations be calculated, but the proportion of imaging use in all patients could be used where individual GP training to use LBP codes is not practical.

### Suggested future modifications to methods

This study identified a number of challenges that need to be resolved to improve the feasibility of undertaking a fully powered cluster randomised controlled trial. GP recruitment may be improved by prioritising in-person recruitment (e.g. at conferences, clinical practice meetings). Where in-person recruitment is not practical, recruiting through current healthcare infrastructure (e.g. local healthcare districts) may provide the large cohort of clinics that would be required to meet sample size targets. Recruitment should be monitored to ensure an adequate spread in GP baseline demographics and block randomisation may be necessary for baseline characteristics such as frequency of imaging referral, years in clinical practice, and beliefs about imaging to ensure even spread between intervention and control groups [32]. Dedicated time for individual GP training, while resource intensive, needs to be considered to ensure all GPs will be able to attend the training session. The provision of online training recordings should be used as a reminder, rather than a replacement, of the in-person training session.

## Conclusion

This pilot cluster randomised controlled trial demonstrated the feasibility of many aspects of a full-scale effectiveness study, while also identifying a number of challenges that need to be resolved. Recommendations related to GP recruitment, study compliance, data collection, and outcome measures were made to increase the success of a future trial.

## Supplementary Information

**Additional file 1.** Completed Consort extension for pilot and feasibility trials checklist.

**Additional file 2.** Outline of the training session for GPs in both intervention and control groups.

**Additional file 3.** Copy of the baseline questionnaire for GPs.

**Additional file 4.** Copy of the post-study interview questions for GPs.

**Additional file 5.** Summary of GP qualitative responses for Aim 2.

**Additional file 6.** Individual GP and monthly summary imaging data.

## Data Availability

The datasets generated during and/or analysed during the current study are available from the corresponding author on reasonable request.

## References

[1] Chou R, Deyo RA, Jarvik JG (2012). Appropriate use of lumbar imaging for evaluation of low back pain. Radiol Clin N Am.

[2] Jenkins HJ, Downie AS, Maher CG, Moloney NA, Magnussen JS, Hancock MJ (2018). Imaging for low back pain: is clinical use consistent with guidelines? A systematic review and meta-analysis. Spine J.

[3] Downie A, Hancock M, Jenkins H, Buchbinder R, Harris I, Underwood M (2020). How common is imaging for low back pain in primary and emergency care? Systematic review and meta-analysis of over 4 million imaging requests across 21 years. Br J Sports Med.

[4] Karel YH, Verkerk K, Endenburg S, Metselaar S, Verhagen AP (2015). Effect of routine diagnostic imaging for patients with musculoskeletal disorders: A meta-analysis. Eur J Intern Med.

[5] Lemmers G, van Lankveld W, Westert G, van der Wees P, Staal J. Imaging versus no imaging for low back pain: a systematic review, measuring costs, healthcare utilization and absence from work. Eur Spine J. 2019;28(5):937–50. 10.1007/s00586-019-05918-1 30796513

[6] Jenkins HJ, Hancock MJ, French SD, Maher CG, Engel RM, Magnussen JS (2015). Effectiveness of interventions designed to reduce the use of imaging for low-back pain: a systematic review. CMAJ.

[7] Slade SC, Kent P, Patel S, Bucknall T, Buchbinder R (2016). Barriers to primary care clinician adherence to clinical guidelines for the management of low back pain: a systematic review and meta-synthesis of qualitative studies. Clin J Pain.

[8] Jenkins HJ, Moloney NA, French SD, Maher CG, Dear BF, Magnussen JS (2018). Using behaviour change theory and preliminary testing to develop an implementation intervention to reduce imaging for low back pain. BMC Health Serv Res.

[9] Simula AS, Jenkins HJ, Holopainen R, Oura P, Korniloff K, Häkkinen A (2019). Transcultural adaption and preliminary evaluation of “understanding low back pain” patient education booklet. BMC Health Serv Res.

[10] Matowe L, Ramsay C, Grimshaw J, Gilbert F, Macleod M, Needham G (2002). Effects of mailed dissemination of the royal college of radiologists' guidelines on general practitioner referrals for radiography: a time series analysis. Clin Radiol.

[11] French S, McKenzie J, O'Connor D, Grimshaw J, Mortimer D, Francis J (2013). Evaluation of a theory-informed implementation intervention for the management of acute low back pain in general medical practice: The IMPLEMENT cluster randomised trial. PLoS One.

[12] Schectman J, Schroth S, Verme D, Voss J (2003). Randomized controlled trial of education and feedback for implementation of guidelines for acute low back pain. J Gen Intern Med.

[13] Page MJ, French SD, McKenzie JE, O'Connor DA, Green SE (2011). Recruitment difficulties in a primary care cluster randomised trial: investigating factors contributing to general practitioners' recruitment of patients. BMC Med Res Methodol.

[14] Perkins D, Harris MF, Tan J, Christl B, Taggart J, Fanaian M (2008). Engaging participants in a complex intervention trial in Australian General Practice. BMC Med Res Methodol.

[15] Fritz JM, Brennan GP, Hunter SJ, Magel JS (2013). Initial management decisions after a new consultation for low back pain: implications of the usage of physical therapy for subsequent health care costs and utilization. Arch Phys Med Rehabil.

[16] Webster B, Bauer AS, Choi Y, Cifuentes M, Pransky G (2013). Iatrogenic consequences of early MRI in acute work-related disabling low back pain. Spine..

[17] Hirsch JA, Nicola G, McGinty G, Liu RW, Barr RM, Chittle MD (2016). ICD-10: History and Context. Am J Neuroradiol.

[18] McKinn S, Bonner C, Jansen J, McCaffery K (2015). Recruiting general practitioners as participants for qualitative and experimental primary care studies in Australia. Austr J Prim Health.

[19] Eldridge SM, Chan CL, Campbell MJ, Bond CM, Hopewell S, Thabane L (2016). CONSORT 2010 statement: extension to randomised pilot and feasibility trials. BMJ..

[20] Jenkins H. Low back pain management booklet Macquarie Univeristy. Sydney: Macquarie University; 2020. Available from: https://tinyurl.com/lowbackpaineducation.

[21] Almeida M, Saragiotto B, Richards B, Maher CG (2018). Primary care management of non-specific low back pain: key messages from recent clinical guidelines. Med J Austr.

[22] NSW Agency for Clinical Innovation (2016). Management of people with acute low back pain: model of care.

[23] Maher C, Underwood M, Buchbinder R (2017). Non-specific low back pain. Lancet..

[24] Gale NK, Heath G, Cameron E, Rashid S, Redwood S (2013). Using the framework method for the analysis of qualitative data in multi-disciplinary health research. BMC Med Res Methodol.

[25] Britt H, Miller G, Bayram C, Henderson J, Harrison C, Pan Y (2016). A decade of Australian general practice activity 2006-07 to 2015-16, General pactice series no. 41.

[26] Williams C, Maher C, Hancock M, McAuley J, McLachlan A, Britt H (2010). Low back pain and best practice care. Arch Intern Med.

[27] Buchbinder R, Staples M, Jolley D (2009). Doctors With a Special Interest in Back Pain Have Poorer Knowledge About How to Treat Back Pain. Spine (Phila Pa 1976).

[28] Cheung A, Weir M, Mayhew A, Kozloff N, Brown K, Grimshaw J (2012). Overview of systematic reviews of the effectiveness of reminders in improving healthcare professional behavior. Syst Rev.

[29] Morgan T, Wu J, Ovchinikova L, Lindner R, Blogg S, Moorin R (2019). A national intervention to reduce imaging for low back pain by general practitioners: a retrospective economic program evaluation using Medicare Benefits Schedule data. BMC Health Serv Res.

[30] Miller G, Valenti L, Charles J (2006). Use of diagnostic imaging in Australian general practice. Austr Fam Phys.

[31] Bolzern J, Mnyama N, Bosanquet K, Torgerson DJ (2018). A review of cluster randomized trials found statistical evidence of selection bias. J Clin Epidemiol.

[32] Campbell MJ (2019). Cluster randomised trials. Med J Austr.

