# Supplementary material for: Feasibility of testing the effectiveness of a theory-informed intervention to reduce imaging for low back pain: a pilot cluster randomised controlled trial
Source: Pilot Feasibility Stud. 2022 Dec 9;8:249. doi: 10.1186/s40814-022-01216-8 (PMC9733261; doi:10.1186/s40814-022-01216-8)
Supplement: Supplementary file 2 — Additional file 2. Outline of the training session for GPs in both intervention and control groups. [file 40814_2022_1216_MOESM2_ESM.pdf]

GP training outline

Consent and Medicare data release

Ensure that consent has been provided and that the Medicare data release form has been signed before proceeding

Baseline questionnaire (all GPs)

Before the training session starts ask the GP to complete the baseline questionnaire

Study processes (all GPs)

- Run through the study flow chart
- Demonstrate how to use low back pain codes on electronic medical record software
- Enter a code containing ‘low back’, ‘lumbar’, ‘lumbosacral’, or ‘sciatica’ in the ‘Reason for visit’ field for any patients presenting with low back pain during the study period
- Manage patients as usual (control group) or using the booklet (intervention group)

Training to use low back pain management booklet (intervention group only)

Run through practitioner training powerpoint presentation, including:

- Introduction to the booklet and why it was developed
- How the booklet was developed
- Likely benefits of using the booklet
- Overview of low back pain diagnosis and management, including guideline recommendations
- How to use the different elements of the booklet during a clinical consult
- Hardcopy and online options

Provide hardcopy and PDF copies of the booklet and a pre-recorded video of the training session for future reference.
