# Supplementary material for: Feasibility of testing the effectiveness of a theory-informed intervention to reduce imaging for low back pain: a pilot cluster randomised controlled trial
Source: Pilot Feasibility Stud. 2022 Dec 9;8:249. doi: 10.1186/s40814-022-01216-8 (PMC9733261; doi:10.1186/s40814-022-01216-8)
Supplement: Supplementary file 3 — Additional file 3. Copy of the baseline questionnaire for GPs. [file 40814_2022_1216_MOESM3_ESM.pdf]

Baseline Questionnaire

Provider ID: _______________________

1. Gender: ❒ Male

   ❒ Female

2. Year of graduation from medical degree: _________________

3. Number of years practicing as a general practitioner: _________________

4. Do you have a special interest in back pain as a general practitioner?

   ❒ Yes

   ❒ No

5. Have you done any continuing education in back pain in the last 2 years?

   ❒ Yes

   ❒ No

6. We are interested in what you think about managing low back pain as a general practitioner. Please indicate your views below by circling the appropriate number on the scale.

1=COMPLETELY DISAGREE 5= COMPLETELY AGREE

	COMPLETELY DISAGREE				COMPLETELY AGREE
A. I feel confident in my ability as a general practitioner to manage patients with low back pain	1	2	3	4	5
B. I feel pressure from patients to refer for imaging (X-rays, CT, MRI) for low back pain	1	2	3	4	5
C. Imaging (X-rays, CT, MRI) of the lumbar spine is useful in the workup of patients with acute low back pain	1	2	3	4	5
D. I am likely to order imaging (X-rays, CT, MRI) for acute low back pain	1	2	3	4	5
