# Supplementary material for: Feasibility of testing the effectiveness of a theory-informed intervention to reduce imaging for low back pain: a pilot cluster randomised controlled trial
Source: Pilot Feasibility Stud. 2022 Dec 9;8:249. doi: 10.1186/s40814-022-01216-8 (PMC9733261; doi:10.1186/s40814-022-01216-8)
Supplement: Supplementary file 4 — Additional file 4. Copy of the post-study interview questions for GPs. [file 40814_2022_1216_MOESM4_ESM.pdf]

Post study Questionnaire

Practitioner ID: _______________________

<u>All practitioners</u>

1. On a scale of 0 to 5, where 0 is you didn't use them at all and 5 is you used them all the time, please rate your ability to use the codes for low back pain within the practice management software, as directed in the initial training session.

2. Why were you able or not able to code patients for low back pain?

3. What codes did you most commonly use?

<u>Intervention group practitioners</u>

1. On a scale of 0 to 5, where 0 is you didn't use it at all and 5 is you used it all the time, please rate your ability to use the provided intervention within clinical practice, as directed in the initial training session.

2. Why did you use or not use the intervention?

3. Did you use the hardcopy or digital version of the intervention, or both, and why?

4. On a scale of 0 to 5, where 0 is completely unimpressed and 5 is very impressed, what was your overall impression of the hardcopy booklet? Why?

5. On a scale of 0 to 5, where 0 is completely unimpressed and 5 is very impressed, what was your overall impression of the digital version of the booklet? Why?

6. On a scale of 0 to 5, where 0 is not useful at all and 5 is very useful, please rate how useful you found the intervention to help manage patients with low back pain.

7. Why did you find the intervention useful or not useful?

8. On a scale of 0 to 5, where 0 is not useful at all and 5 is very useful, please rate how useful you found the initial training session to instruct you on how to use the intervention in practice.

9. Why did you find the training session useful or not useful?

10. Are there any improvements to the intervention or the training session that you can think of?

11. Do you have any further comments?
