# Supplementary material for: Feasibility of testing the effectiveness of a theory-informed intervention to reduce imaging for low back pain: a pilot cluster randomised controlled trial
Source: Pilot Feasibility Stud. 2022 Dec 9;8:249. doi: 10.1186/s40814-022-01216-8 (PMC9733261; doi:10.1186/s40814-022-01216-8)
Supplement: Supplementary file 5 — Additional file 5. Summary of GP qualitative responses for Aim 2. [file 40814_2022_1216_MOESM5_ESM.pdf]

Additional file 5

Table: Summary of GP qualitative responses for Aim 2

GP ID	Use of LBP codes (all GPs)			Intervention training session (intervention group GPs only)			
	**Frequency of use***	**Barriers to using codes**	**Facilitators to using codes**	**Attended training (Y/N)**	**Usefulness of training session#**	**Most useful elements**	**Recommendations for improvement**
**Intervention group GPs**							
GP1	It was all the time 5 out of 5		Because the patient come in with the complaint of the pain. So I just put in the front of my mind and useful to use them.	Y	5/5	Just the face to face helps quite a lot. It was really helpful because sometimes you need to ask a question and sometimes you can't use mobile phone text message or email. So in face to face discussion helped a lot.	More information. The quality of the chair for the patient. Compared to before for the back pain.
GP2	I would like to say 5, but I'm not 100% that I actually did so I'm going to go with 4.	Just pure in a rush		Y	I suppose it would have been a 4 or a 5	It was not so much that there was new information as such. But it was more so consolidating things which is always useful. Just to summarise what the latest evidence is on management and things. Its good to know oh yes I'm doing things right there.	it was fine. I think the difficulty isn't giving people the information its actually getting people to go away and actually do it.
GP14	5/5 I used it all the time I believe so		They are standard codes that we already use.	Y	Without the training I wouldn't know what to do, so it would have to be a 5 right. You need it.	Yes to deliver the information.	I think maybe, because of the length of time and I am sure COVID had something to do with it. Maybe just an update a month in to just see if there are any kinks or questions just a that's what I would of done, you know quick phone call to say how's it going? If you did do it and I don't remember I apologise. But I don't remember.
GP15	I use 5 out of 5. So I always use the computer generated		Because I run the practice and all the general practice division they encourage us to use the code and I have to tell	Y	I can't remember very much to be honest.		If you explain your study. I mean the study is there. I don't know. I think you did mention that you can use the digital one but we didn't actually load it into

	code. That's what you want.		my doctors to do that. And they don't do that sometimes, so I have to set an example. Unless something that I really can't find, but for back pain, sciatica and all those types of things I always use the code.		It was very quick I think. I would say the initial instruction was not that great. 2 out of 5 I would say.		the computer. Then I forgot all about it and thought oh no there is a digital one to.<br>I think you need to set aside more time to train the Drs maybe you know, but we sort of know the gist of it anyway. I don't know how much more we can be trained in a sense. Maybe just a reminder you know that mind be helpful. I know you have reminders on email and the other thing is that the COVID-19 thing really did make a difference and a lot of people didn't turn up.
**Control group GPs**							
GP3	4/5	If it was related to for example something as in renal stones or a urinary tract infection then I was in doubt whether use it [LBP codes] or not	to use the codes if they had a real issue with LBP, yes I did use LBP [codes]. so when I can say whether it is related to a LBP issue then I did use the code LBP	Y (training to use LBP codes only)			
GP4	5/5		Because I got that in my mind that I was participating in this research, so I wanted to do my job.	Y (training to use LBP codes only)			
GP5	3/5, used them most of the time but not all of the time	You forget, when you see patients and the next patient waiting.<br>There is only one code we can use in our system. If patients presenting with a few complaints that include low back pain, didn't use them then, because the main issue we need to consider.	When they come with the one issue not multiple issues. If they have worse low back pain than others then you are more likely to use it.	Y (training to use LBP codes only)			
GP7	I would say 5 in terms of when I needed to use them it was very simple and I would of done it every time		I'm a bit of a stickler for detail and data in terms of the reason for visit, which is what we are talking about is one thing that I always make sure that I do properly. And so it is something that I would do all the time.	Y (training to use LBP codes only)			
GP10	2/10	the biggest issue was that I simply did not have enough patients coming in with	So what made it easy was it was pretty straight forward.	Y (training to use LBP			

		lower back pain, because for a fair amount of time since joining the group I have been on leave. Also seeing far less patients because of COVID. And also only just starting to see patients for things like lower back pain since the introduction of the new teleconferencing item numbers. In terms of patients that I have seen with lower back pain, sometimes I've just forgotten.	Had I taken the time to do it there was nothing difficult about using those codes	codes only)			
GP13	0/10	I just forgot about I would say. I just forgot about the study. So I just didn't think of it.		Y (training to use LBP codes only)			

*Scale of 0-5, 0=didn't use codes at all, 5=used codes all the time

#Scale of 0-5: 0=training not useful at all, 5=training very useful
