# Supplementary material for: Feasibility of testing the effectiveness of a theory-informed intervention to reduce imaging for low back pain: a pilot cluster randomised controlled trial
Source: Pilot Feasibility Stud. 2022 Dec 9;8:249. doi: 10.1186/s40814-022-01216-8 (PMC9733261; doi:10.1186/s40814-022-01216-8)
Supplement: Supplementary file 6 — Additional file 6. Individual GP and monthly summary imaging data. [file 40814_2022_1216_MOESM6_ESM.pdf]

Additional File 6

Table: Individual GP and monthly summary imaging data

	Pre-intervention period																			
	Month 1					Month 2					Month 3					Month 4				
GP	LBP Patients	Total patient visits	Lumbar imaging	Imaging proportion LBP patients (%)	Imaging proportion /1000 patients	LBP Patients	Total patient visits	Lumbar imaging	Imaging proportion LBP patients (%)	Imaging proportion /1000 patients	LBP Patients	Total patient visits	Lumbar imaging	Imaging proportion LBP patients (%)	Imaging proportion /1000 patients	LBP Patients	Total patient visits	Lumbar imaging	Imaging proportion LBP patients (%)	Imaging proportion /1000 patients
GP1	16	795	11	68.8	13.8	24	922	5	20.8	5.4	17	605	2	11.8	3.3	14	649	9	64.3	13.9
GP2	9	342	2	22.2	5.8	13	483	6	46.2	12.4	10	435	3	30.0	6.9	12	366	3	25.0	8.2
GP3	0	ND	0	0.0	ND	0	ND	0	0.0	ND	0	ND	0	0.0	ND	0	ND	0	0.0	ND
GP4	5	ND	1	20.0	ND	6	ND	2	33.3	ND	3	ND	1	33.3	ND	4	ND	1	25.0	ND
GP5	2	ND	0	0.0	ND	0	ND	0	0.0	ND	1	ND	1	100.0	ND	0	ND	0	0.0	ND
GP7	3	127	1	33.3	7.9	2	97	0	0.0	0.0	2	106	0	0.0	0.0	4	110	0	0.0	0.0
GP10	0	119	0	0.0	0.0	0	94	0	0.0	0.0	0	63	0	0.0	0.0	1	109	0	0.0	0.0
GP13	0	79	0	0.0	0.0	0	75	0	0.0	0.0	0	60	0	0.0	0.0	0	74	0	0.0	0.0
GP14	14	369	8	57.1	21.7	10	472	3	30.0	6.4	4	279	1	25.0	3.6	1	242	0	0.0	0.0
GP15*	8	427	9	112.5	21.1	4	275	0	0.0	0.0	2	295	2	100.0	6.8	7	456	1	14.3	2.2
**Total**	**57**	**2258**	**32**	**56.1**	**14.2**	**59**	**2418**	**16**	**27.1**	**6.6**	**39**	**1843**	**10**	**25.6**	**5.4**	**43**	**2006**	**14**	**32.6**	**7.0**

	Intervention period																			
	Month 5					Month 6					Month 7					Month 8				
GP	LBP Patients	Total patient visits	Lumbar imaging	Imaging proportion LBP patients (%)	Imaging proportion /1000 patients	LBP Patients	Total patient visits	Lumbar imaging	Imaging proportion LBP patients (%)	Imaging proportion /1000 patients	LBP Patients	Total patient visits	Lumbar imaging	Imaging proportion LBP patients (%)	Imaging proportion /1000 patients	LBP Patients	Total patient visits	Lumbar imaging	Imaging proportion LBP patients (%)	Imaging proportion /1000 patients
GP1	21	862	7	33.3	8.1	20	547	6	30.0	11.0	18	835	4	22.2	4.8	22	849	14	63.6	16.5

GP2	13	378	1	7.7	2.6	16	442	5	31.3	11.3	17	434	2	11.8	4.6	8	323	3	37.5	9.3
GP3	0	ND	0	0.0	ND	0	ND	0	0.0	ND	0	ND	0	0.0	ND	0	ND	0	0.0	ND
GP4	3	ND	0	0.0	ND	5	ND	0	0.0	ND	7	ND	0	0.0	ND	7	ND	3	42.9	ND
GP5	0	ND	0	0.0	ND	4	ND	4	100.0	ND	2	ND	2	100.0	ND	1	ND	1	100.0	ND
GP7	0	110	0	0.0	0.0	0	41	0	0.0	0.0	2	108	0	0.0	0.0	6	91	0	0.0	0.0
GP10	0	89	1	1/0	11.2	1	105	0	0.0	0.0	1	104	0	0.0	0.0	0	71	0	0.0	0.0
GP13	2	22	0	0.0	0.0	0	61	2	2/0	32.8	0	67	0	0.0	0.0	1	68	0	0.0	0.0
GP14	8	449	1	12.5	2.2	5	527	2	40.0	3.8	4	774	1	25.0	1.3	2	471	0	0.0	0.0
GP15	5	272	0	0.0	0.0	3	432	2	66.7	4.6	3	574	8	266.7	13.9	8	435	12	150.0	27.6
**GP15**	**52**	**218<br>2**	**10**	**19.2**	**4.6**	**54**	**2155**	**21**	**38.9**	**9.7**	**54**	**2896**	**17**	**31.5**	**5.9**	**55**	**2308**	**33**	**60.0**	**14.3**

Legend: Monthly counts of LBP patients extracted from clinic electronic medical record; Monthly counts of total patient visits and lumbar imaging extracted from Medicare data; ND: Data not available; *Number of LBP patients lower than number of lumbar images for GP15 in months 1, 7, and 8. This may be due to lack of coding for LBP patients, or an imaging referral from a previous month being used in this time period
